# AMP-Activated Protein (AMPK) in Pathophysiology of Pregnancy Complications

**DOI:** 10.3390/ijms19103076

**Published:** 2018-10-09

**Authors:** Asako Kumagai, Atsuo Itakura, Daisuke Koya, Keizo Kanasaki

**Affiliations:** 1 Department of Diabetology and Endocrinology, Kanazawa Medical University, Uchinada, Ishikawa 920-0293, Japan; a-kumagai@juntendo.ac.jp (A.K.); koya0516@kanazawa-med.ac.jp (D.K.); 2 Department of Obstetrics and Gynecology, Juntendo University, Bunkyo-ku, Tokyo 113-0033, Japan; a-itakur@juntendo.ac.jp; 3 Division of Anticipatory Molecular Food Science and Technology, Medical Research Institute, Kanazawa Medical University, Uchinada, Ishikawa 920-0293, Japan

**Keywords:** pregnancy, catechol-*O*-methyltransferase, 2-methoxyestradiol, preeclampsia, gestational diabetes mellitus

## Abstract

Although the global maternal mortality ratio has been consistently reduced over time, in 2015, there were still 303,000 maternal deaths throughout the world, of which 99% occurred in developing countries. Understanding pathophysiology of pregnancy complications contributes to the proper prenatal care for the reduction of prenatal, perinatal and neonatal mortality and morbidity ratio. In this review, we focus on AMP-activated protein kinase (AMPK) as a regulator of pregnancy complications. AMPK is a serine/threonine kinase that is conserved within eukaryotes. It regulates the cellular and whole-body energy homeostasis under stress condition. The functions of AMPK are diverse, and the dysregulation of AMPK is known to correlate with many disorders such as cardiovascular disease, diabetes, inflammatory disease, and cancer. During pregnancy, AMPK is necessary for the proper placental differentiation, nutrient transportation, maternal and fetal energy homeostasis, and protection of the fetal membrane. Activators of AMPK such as 5-Aminoimidazole-4-carboxamide ribonucleotide (AICAR), resveratrol, and metformin restores pregnancy complications such as gestational diabetes mellitus (GDM), preeclampsia, intrauterine growth restriction, and preterm birth preclinically. We also discuss on the relationship between catechol-*O*-methyltransferase (COMT), an enzyme that metabolizes catechol, and AMPK during pregnancy. It is known that metformin cannot activate AMPK in COMT deficient mice, and that 2-methoxyestradiol (2-ME), a metabolite of COMT, recovers the AMPK activity, suggesting that COMT is a regulator of AMPK. These reports suggest the therapeutic use of AMPK activators for various pregnancy complications, however, careful analysis is required for the safe use of AMPK activators since AMPK activation could cause fetal malformation.

## 1. Introduction

Although the global maternal mortality ratio has been consistently reduced, in 2015 there were still 303,000 maternal deaths worldwide, 99% of which occurred in developing countries {Alkema, 2016 #3}. Among those countries, accelerated reduction of the maternal mortality rate was observed in countries that improved their transportation systems, health facilities (including the number of free-standing health facilities), training of health-care providers as well as education [1]. The proper prenatal care has a significant impact on educating the expectant mothers and their family members as well as the prevention of pregnancy complications. Pregnancy complications have two aspects, one is maternal complications such as gestational diabetes mellitus (GDM), and preeclampsia, the other is fetal complications such as intrauterine growth restriction (IUGR), and the risk of preterm birth (PTB). Many of those complications have previously reported to be associated with AMP-activated protein kinase (AMPK), a stress-induced enzyme.

AMPK is a serine/threonine kinase that is conserved within eukaryotes. It is formed of a heterotrimeric complex, which consists of a catalytic α subunit and regulatory β, γ subunits. Two isomers exist for the α and β subunits, (α1, α2), (β1, β2), and three isomers for the γ subunit (γ1, γ2, γ3), and the combinations are highly tissue specific [2,3,4,5]. Each subunit isoform is encoded by specific gene such as PRKAA1 for AMPK α1, and PRKAB1 for AMPK β1. AMPK regulates the cellular and whole-body energy homeostasis under stress condition. When cells are stressed, consumption of ATP increases, subsequently resulting in elevation of the AMP/ATP ratio [2]. This elevation of the AMP/ATP ratio activates AMPK via phosphorylation of αThr^172^ [3]. Activated AMPK shifts the cell metabolism from anabolism to catabolism to increase the cellular ATP concentration [2]. AMPK is also activated by intracellular calcium and oxidant signaling as well as extracellular signaling by hormones and cytokines [4]. The functions of AMPK are diverse and include glucose and lipid metabolism, protein synthesis, mitochondrial biogenesis, redox reaction, anti-inflammation, anti-oxidative stress, anti-apoptosis, and nitric oxide synthesis [5,6,7]. Due to its many functions as a regulator of energy balance, many disorders have been known to correlate with AMPK such as cardiovascular disease, diabetes, inflammatory disease, and cancer [5,8,9].

Activated AMPK exists in placental tissue and the uterine artery of humans and mice, contributing to placental differentiation and fetal growth [10]. On the other hand, AMPK activity is decreased during gestation in the hypothalamus, the brain area that maintains whole body energy balance. In non-pregnant rats, when energy balance was negative, AMPK activation increased, as well as fatty acid synthase (FAS) and the anorectic signal, malonyl-CoA, decreased in the hypothalamus to induce food intake. However, in pregnant rats, hypothalamic AMPK activation and FAS expression decreased and malonyl-CoA increased although food intake is enhanced, suggesting resistance to anorectic signals during gestation [11] (Table 1). Pregnancy changes the metabolic balance to maintain the energy demand for the embryonic growth. In this review, we focus on the relationship between AMPK activity and pregnancy complications.

## 2. Intrauterine Growth Restriction (IUGR)

IUGR is one of the leading causes of perinatal mortality [26,27]. Except for genetic factors, fetal growth depends on maternal and utero-placental factors. Maternal factors are represented by maternal nutrition and hypoxia. Caloric restriction decreases maternal circulating insulin-like growth factor 1 (IGF-1), leptin, insulin, and increases cortisol in humans and animals. These maternal hormones are known as regulators for placental nutrient transport. In human IUGR, the activity of amino acid transporter system A (system A) is low in the microvillous membrane of the syncytiotrophoblast [28,29]. In vivo experiments using rodent and sheep suggested that IGF-1, leptin and insulin stimulated the activity of system A, whereas cortisol reduced placental nutrient transport [30,31]. This activation of system A by maternal hormones is known to be mediated by activated mammalian target of rapamycin (mTOR) signaling. mTOR induces cell growth and proliferation in a nutrition rich environment by sensing nutrient availability [32]. In human trophoblast cells, mTOR positively correlated with system A activation [28,32]. Human, baboon, and rodent studies also supported that calorie restriction reduced placental mTOR activity, resulting in decreasing nutrient transport and increasing the risk of IUGR. AMPK is an inhibitor of mTOR, however, baboon placental AMPK activity was unchanged in caloric restriction studies [28,32,33].

Sufficient oxygen supply is also required for nutrient transport [34]. Women living at higher altitudes have a decreased ability for placental nutrition exchange compared to women living at lower altitudes [35]. Under maternal hypoxia, utero-placental oxygen supply decreases regardless of defensive adaptation against low oxygen such as fetal polycythemia, resulting in a higher rate of IUGR [36,37]. Among utero-placental factors, uterine factors such as uterine malformation, uterine myoma, and adenomyosis directly obstruct uterine expansion and placental factors obstruct nutrient transport due to improper placental differentiation or reduced blood supply. Blood supply is essential for the fetus to acquire nutrients through the placenta. In vivo experiment of impaired utero-placental blood flow by ligating the uterine artery decreased the glucose and amino acid transportation in rats [12] (Table 1). The cellular mTOR signaling was inhibited under hypoxic condition via activation of AMPK in HEK293 cells and mouse embryonic fibroblasts (MEF) cells [38]. Indeed, placental mTOR expression is inhibited in pregnant women at high altitude who are known to have less ability for placental nutrient transport [39]. In vivo study revealed that activated AMPK increased uterine artery blood flow velocity either by inhibition of vasoconstriction prostanoids or by increasing nitric oxide production [12]. Resveratrol, the natural polyphenol that is found in grapes, cranberries, and red wine, is known to activate AMPK. Administration of resveratrol to the pregnant mice under severe hypoxic condition improved fetal survival and fetal growth [13] (Table 1). AMPK knockdown SM10 cells (mouse trophoblast progenitor cells), created by infecting lentivirus containing AMPK α1/2 shRNA, were shown to have less ability of cell growth (<50%). In addition, TGF-β induced SM10 cell differentiation was inhibited by AMPK knockdown. In terms of nutrient transport, AMPK knockdown reduced glucose transport by inhibiting expression of glucose transporter 3 (GLUT3) in SM cells. Immunohistochemistry revealed that the normal cellular localization of GLUT3 was mainly on the cell surface, indicating the proper glucose transportation, whereas AMPK knockdown cells exhibited GLUT3 localized near the nucleus [10].

These reports clearly demonstrate that AMPK activation is important for nutrient transport both by increasing uterine blood flow and increasing glucose receptor on cell surface.

## 3. Gestational Diabetes Mellitus (GDM)

GDM is defined as glucose intolerance that is first diagnosed after conception. The frequency of GDM differs depending on the ethnicity, but it is reported as 2–5% worldwide [15]. Obesity and family history of diabetes are the risk factors of GDM. GDM increases the risk of hypertensive disorders of pregnancy (HDP), large for gestational age, shoulder dystocia, nerve palsies, neonatal hypoglycemia, hyperbilirubinemia, and polycythemia. More than 90% of GDM resolves soon after the delivery, however, the long-term morbidity of type-2 diabetes is seven-times more frequent than women without GDM [40]. Prevention and treatment of GDM is important both for mothers and their offspring.

During pregnancy, the placenta produces hormones such as human placental lactogen (hPL), which decreases maternal insulin sensitivity and maternal glucose utilization to transport sufficient glucose through the placenta to the fetus. GDM develops when maternal insulin production by pancreatic β cells does not match with the insulin sensitivity of the organs [41,42].

In the first trimester, the development of the placenta induces drastic alternations in the trophoblast environment. Placenta and trophoblast are grown under a hypoxic environment in the beginning of gestation, however, once the spiral artery develops, the oxygen level increases, which results in an increase in the oxidative stress [43]. If GDM develops in the first trimester, the embryo is damaged both by oxidative and hyperglycemic stress, resulting in growth restriction of the placental embryonic unit [44,45]. In the mid and late trimester, fetal demand of oxygen is higher in GDM due to the enhanced metabolism by fetal high insulin levels. To meet the demand from the fetus, the placenta increases its volume and promotes angiogenesis to supply more oxygen, and erythropoiesis along with fetus growth [46,47]. Similar to in the first trimester, high oxygen supply induces oxidative stress as well as hyperglycemic stress in the fetal environment during the mid-late trimester. The maternal hyperglycemic environment enriches cellular ATP which inactivates AMPK and activates mTOR in the liver of humans, and mice [14,16] (Table 1). In the placenta, gene expression of AMPK is suppressed, and m-TOR activation is enhanced in GDM women [48] (Table 1).

There were several reports that AMPK activation by chemical compounds, such as resveratrol, 5-Aminoimidazole-4-carboxamide ribonucleotide (AICAR), and metformin, cured GDM preclinically [16,49,50]. Resveratrol was known to reduce high-glucose induced oxidative stress by activation of AMPK in type-2 diabetic animal models [51]. In GDM model mice, resveratrol increased phosphorylated AMPK (p-AMPK) in the maternal liver and lowered the maternal insulin resistance and the fetal body weight while increasing the fetal survival rate via increasing the activity of glucose-6-phosphatase in both mother and offspring [16]. Metformin is known to decrease hepatic glucose production, to increase glucose uptake in peripheral tissues, and to lower plasma triglyceride and free fatty acids. Metformin activates AMPK directly by phosphorylating Th172 on the α subunit, or indirectly by inhibiting mitochondrial complex I which increases the AMP/ATP ratio [52,53]. Active AMPK in skeletal muscle enhanced insulin-stimulated GLUT4 expression to increase glucose uptake, while in the hepatic tissue, gluconeogenic genes were inhibited by active AMPK [54,55]. Active AMPK also stimulated glucose uptake in skeletal muscle independent of insulin [56,57]. One concern about taking medication by pregnant women is placental transportability. It is known that embryonic AMPK activation stimulated by hyperglycemic and oxidative stress in GDM patients causes neural tube defect (NTD) through inhibiting the expression of pax3, an essential gene for neural tube closure [58]. Ex vivo study showed that metformin-treated mouse embryonic stem cell-derived neural progenitor cells increased activated AMPK and reduced pax3 expression [59]. However, in in vivo studies, metformin did not increase active AMPK in the embryos of pregnant mice [59]. It was reported that metformin did not pass through to the embryo due to insufficient expression of the metformin transporter, *Oct3/Slc22*, on the embryo during the period of organogenesis [59,60]. On the other hand, the placenta had sufficient expression of *Oct3/Slc22*, and some clinical studies reported that there were no significant differences in incidence of LGA, mean birthweight, and neonatal morbidity with metformin treatment and insulin treatment of GDM women [61,62]. Further study is expected for the safety of metformin in GDM. 

In summary, activation of maternal AMPK ameliorates maternal diabetic features and normalizes fetal growth as a result. The safety of AMPK activators should be further studied. 

## 4. Preeclampsia

HDP affects about 10% of all pregnancies in the world [63]. HDP is a group of four diseases, gestational hypertension, chronic hypertension, preeclampsia, and eclampsia. Among them, preeclampsia is the leading cause of maternal and perinatal mortality and morbidity. Preeclampsia is defined as development of new-onset hypertension with proteinuria after 20 weeks of gestation. The progression of preeclampsia results in placental insufficiency, which induces IUGR, and maternal organ dysfunction such as HELLP syndrome, a complication of pregnancy characterized by hemolysis, elevated liver enzymes, and low platelet counts, and eclampsia [63]. Although pathogenesis of preeclampsia is only partially understood, failure in placentation during the early stage of pregnancy has been thought to be a crucial factor that exposes the placenta and embryo to oxidative and inflammatory stress [64,65]. The mal-placentation causes a hypoxic environment, which induces angiogenic imbalance (vascular endothelial growth factor; VEGF< soluble fms-like tyrosine kinase-1; sFlt-1) and hypertension [64,66]. As mentioned in the IUGR section, AMPK activation is required for placental differentiation and vasodilation of uterine artery blood flow. Treatment of hypertension with AICAR restored blood pressure (BP) and angiogenic balance (VEGF > sFlt-1) in rats [19] (Table 1). Metformin exerted the reduction of sFlt-1 secretion on endothelial cells, villous cytotrophoblast cells, and preterm preeclamptic placental villous explants in primary human tissues [17] (Table 1). Metformin also improved vasorelaxation of human omental blood vessels which were cultured in placental villous explants obtained from patients with severe early onset preeclampsia, to the level of vessels cultured in normal media. Metformin also restored the outgrowth of omental vessel rings which was reduced when it was treated solely with sFlt-1 [17] (Table 1). Another study indicated that in preeclamptic maternal serum, p-AMPK was positively correlated with the severity of preeclampsia and BP, while it was negatively correlated with gestational week at delivery and birth weight [18] (Table 1). In summary, lack of AMPK induces mal-placentation, which results in angiogenic imbalance. The increase in serum AMPK in severely preeclamptic women suggests a compensatory mechanism for the angiogenic imbalance. AMPK activators ameliorate the preeclamptic symptoms, which indicates AMPK as a potential therapeutic target of preeclampsia

In the immunological view, imbalance between regulatory T (Treg) cells and Th17 cells is reported in preeclamptic women. Treg cells exhibit immunological tolerance during pregnancy. Th17 cells, on the other hand, induce inflammation. In normal pregnancies, increase in Treg cells and decrease in Th17 cells are found in peripheral blood compared to non-pregnant women [67]. However, in preeclampsia, Treg cells decrease and Th17 cells increase compared to non-pregnant levels [68,69]. AMPK activation restored the normal balance between Treg and Th17 cells and cured such an imbalance. Active AMPK is reported to induce Treg cells development and reduce Th17 cells differentiation, as a result, systemic inflammation improves, and immunological homeostasis is maintained [70].

## 5. Preterm Birth (PTB)

Complications of PTB are the major cause of neonatal deaths, and second leading cause of death among children under five years old. Many of the survived children suffer from lifelong disabilities [71]. Lowering the rate of PTB is in great demand in the world.

The common risk factors of PTB are inflammation and oxidative stress. By generating uterine specific depletion of p53 mice, the model mice of PTB, several studies have found that the activation of mTOR signaling induced decidual senescence during early pregnancy and phosphorylated mTOR increased COX2-derived prostaglandins, which resulted in spontaneous PTB in 50–60% of p53 depleted mice [21] (Table 1). In addition, AMPK activators, metformin and resveratrol, improved the decidual health and the rate of PTB was reduced in PTB model mice [21] (Table 1). In human fetal membranes, AMPK and p-AMPK exist in amnion epithelium, chorionic trophoblasts and decidua. A study reported that p-AMPK was significantly lower in fetal membrane of spontaneous labor at term compared to caesarean delivery at term and that p-AMPK levels in fetal membranes with pre-labor rupture of membrane was significantly lower compared to intact membranes. Preincubation of AMPK activators, AICAR, phenformin, A769662, decreased inflammatory cytokines such as TNF-α, IL-6, IL1-β, IL-8 levels when human fetal membranes were treated with LPS. These data suggested the anti-inflammatory effect of p-AMPK on fetal membranes [20] (Table 1).

## 6. Reprogramming

For decades, many reports have shown that adverse uteroplacental environments have strong associations with metabolic diseases, cardiovascular diseases, skeletal muscle deformity, and cognitive impairments in adult offspring; this concept is known as the developmental origin of health and disease (DOHaD) [72,73]. The DOHaD concept offers a reprogramming strategy that shifts the therapeutic intervention from adulthood to early-life [74]. IUGR rats had higher p-AMPK in hypothalamus regardless of feeding compared to appropriate for gestational age (AGA) rats. It has also been shown that IUGR rats expressed increased orexigenic and decreased anorexigenic mRNA expression in the hypothalamus, resulting in enhanced appetite drive, which contributes to adult obesity [22] (Table 1). On the other hand, there were reports in rats that offspring who were grown under a mal-uteroplacental environment such as mal-nutrition, hyperglycemia, and oxidative stress had reduced hepatic p-AMPK after several weeks from birth [23,24] (Table 1). Many studies have reported that administration of AMPK activators such as resveratrol, metformin and natural polyphenol containing foods (azuki bean, green tea etc.) to the pregnant mice or mice under lactation improved the offspring’s outcome [24,25] (Table 1). Moreover, in rodents, resveratrol applied directly to the offspring also improved their adverse effects of growing under a mal-uteroplacental environment [8,75]. Growth hormone also reversed the dyslipidemia in small for gestational age (SGA) rat offspring grown under mal-nutrition. Hepatic p-AMPK, which was a regulator of lipid and glucose metabolism in the liver, showed no significant difference between SGA rats and AGA rats on neonatal day 1, however, it was significantly lower in SGA rats compared to AGA rats after three weeks from birth. The level of serum triglyceride was also identical between AGA rats and SGA rats at birth, but it significantly increased in the SGA rats at 10 weeks of age. Administration of growth hormone restored the level of hepatic p-AMPK as well as serum triglyceride level and body weight [23] (Table 1). Apparently, maternal environment gives both positive and negative impacts on intra-uterine environment through the placenta, and AMPK activity is key to reversing metabolic imbalance in offspring. Although earlier modification is better, too much activation of AMPK could result in the development of a fetal developmental anomaly such as NTD. Further study is needed for the timing and dose of AMPK activation treatment for both mothers and their offspring.

## 7. Perspective: Catechol-*O*-Methyltransferase and Pregnancy

**Figure 1:**
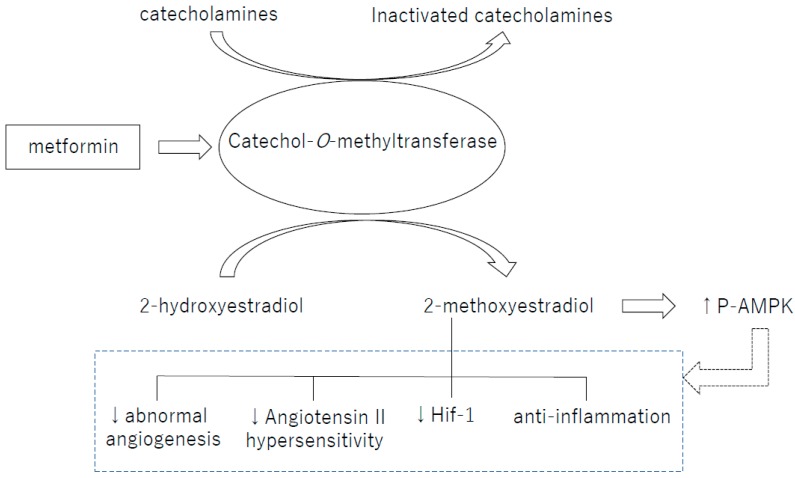
Catechol-*O*-methyltransferase (COMT) is an essential enzyme for the production of 2-methoxyestradiol (2-ME). 2-ME, a metabolite of COMT, induces activation of various molecular pathways, including activation of AMPK. ↑: Increase in activity. ↓: Decrease in activity.

Catechol-*O*-methyltransferase (COMT) is an enzyme that metabolizes catechol such as catecholamines and 2-hydroxyestradiol (2HE), one of the catechol estrogens. 2HE is converted into 2-methoxyestradiol (2-ME) by COMT [76]. In humans, the COMT single-nucleotide polymorphism (SNP) rs4680 (COMT^158Val-Met^) exhibits reduction of enzymatic activity and stability in the Met allele carriers. COMT^158Val-Met^ is associated with many diseases including diabetes, obesity and hypertension [77,78]. Indeed, in preeclamptic women, COMT protein levels and activity have shown to be lower and the COMT-mediated metabolite 2-ME was suppressed in the plasma. As a topic in the AMPK regulation, we would like to introduce our recent findings about the AMPK activation from the view of COMT/2-ME axis (Figure 1). We have recently shown that COMT is an essential enzyme to liver AMPK activity. COMT deficiency either created by a high-fat diet (HFD), COMT inhibitor or siRNA mediated knockdown, induced glucose tolerance defects associated with liver AMPK suppression in mice [79]. Such COMT deficient-associated metabolic defects and suppression of AMPK activation were ameliorated by 2-ME. Metformin recovered the activity of liver AMPK in HFD-treated mice, however, co-administration of the COMT inhibitor suppressed liver AMPK activation [79]. In addition, metformin increased COMT protein expression, suggesting that COMT could be involved in metformin-induced AMPK activation [79]. As well, 2-ME activated AMPK and induced insulin secretion in the cultured insulinoma cell line; MIN-6. The biological significance of such 2-ME-induced AMPK in the insulin secretion is a debatable issue; however in our analysis, AMPK suppression by siRNA in MIN-6 cells abolished 2-ME-induced insulin secretion [79]. A conundrum of this study is that while AMPK is needed for 2-ME-induced insulin secretion; AMPK activator AICAR did not induce insulin secretion in MIN-6 [79]. In the pregnant mice, COMT deficiency is also associated with elevated BP, higher rate of preterm-birth, larger number of fetal wastages, and smaller placentae/decidua [76], all of which are related with lack of p-AMPK. The histological study of COMT deficient placenta showed vascular damage. The elevated BP in COMT deficient mice was explained by increase in angiotensin II receptor type 1 (ATR1) expression, which leads to the hypersensitivity of vascular smooth muscle cells to angiotensin II (AgII). 2-ME suppresses ATR1 expression, and normalizes BP [80]. AgII treatment in mice increased systolic BP and reduced urinary sodium excretion and p-AMPK level in kidney. The AgII antagonist, losartan, and metformin lowered systolic BP and increased urinary sodium excretion and p-AMPK level in kidney in mice [81]. Ex vivo experiments with embryonic rat cardiomyocytes also showed that metformin inhibited AgII-induced upregulation of AgII receptor [82]. Resveratrol was reported to regulate vascular smooth muscle contraction and BP by inhibiting AgII activity [75].

AgII-induced hypertension was ameliorated both by 2-ME and AMPK activators, indicating the close relationship between COMT and AMPK. Further study is required to identify the precise molecular mechanisms of COMT/2-ME axis-associated AMPK activation.

## 8. Conclusions

AMPK maintains the maternal metabolic balance and protects fetal growth from diverse types of stress throughout the pregnancy. Pregnancy complications that are listed in this review have large impacts on maternal or fetal morbidity and mortality. Moreover, they also affect maternal health problems after labor, as well as their offspring’s health problems in adolescence. Supplementation of AMPK activators seems effective for improving both maternal symptoms and fetal growth by restoring the metabolic balance. However, inappropriately activated AMPK in fetus could cause congenital developmental disorders. Thus the timing and the types of AMPK activators should be further studied for safe use.

## Figures and Tables

**Table 1:** Phosphorylated AMP-activated protein kinase (p-AMPK) levels in different organs during pregnancies with complications.

		p-AMPK Levels					
		Maternal					
		Hypothalamus	Liver	Vessel	Placenta	Serum	Fetal Membrane
No complication	human						
	animal model	↓ Ref. [11]					
IUGR	human						
	animal model				↓ Refs. [12,13]		
GDM	human		↓ Ref. [14]		↓ Ref. [15]		
	animal model		↓ Ref. [16]				
Preeclampsia	human			↓ (indirect) Ref. [17]	↓ Ref. [17]	↑ Ref. [18]	
	animal model			↓ (indirect) Refs. [13,19]			
PTB	human						↓ Ref. [20]
	animal model						↓ Ref. [21]
		**Fetal**					
Offspring of complicated pregnancy	human						
	animal model	↑ Ref. [22]	↓ Ref. [23,24,25]				

↑: Increased p-AMPK. ↓: Decreased p-AMPK. Ref: Reference number.
